# Sex Differences in the Morphological and Mechanical Properties of the Achilles Tendon

**DOI:** 10.3390/ijerph18178974

**Published:** 2021-08-26

**Authors:** Xini Zhang, Liqin Deng, Songlin Xiao, Lu Li, Weijie Fu

**Affiliations:** 1 Key Laboratory of Exercise and Health Sciences of Ministry of Education, Shanghai University of Sport, Shanghai 200438, China; 1911516024@sus.edu.cn (X.Z.); 1921516007@sus.edu.cn (L.D.); 1821517025@sus.edu.cn (S.X.); 2 Institute of Sport and Sport Science, University of Freiburg, 79098 Freiburg, Germany

**Keywords:** Achilles tendon, ultrasound, morphological properties, mechanical properties, sex differences

## Abstract

Background: Patients with Achilles tendon (AT) injuries are often engaged in sedentary work because of decreasing tendon vascularisation. Furthermore, men are more likely to be exposed to AT tendinosis or ruptures. These conditions are related to the morphological and mechanical properties of AT, but the mechanism remains unclear. This study aimed to investigate the effects of sex on the morphological and mechanical properties of the AT in inactive individuals. Methods: In total, 30 inactive healthy participants (15 male participants and 15 female participants) were recruited. The AT morphological properties (cross-sectional area, thickness, and length) were captured by using an ultrasound device. The AT force–elongation characteristics were determined during isometric plantarflexion with the ultrasonic videos. The AT stiffness was determined at 50%–100% maximum voluntary contraction force. The AT strain, stress, and hysteresis were calculated. Results: Male participants had 15% longer AT length, 31% larger AT cross-sectional area and 21% thicker AT than female participants (*p* < 0.05). The plantarflexion torque, peak AT force, peak AT stress, and AT stiffness were significantly greater in male participants than in female participants (*p* < 0.05). However, no significant sex-specific differences were observed in peak AT strain and hysteresis (*p* > 0.05). Conclusions: In physically inactive adults, the morphological properties of AT were superior in men but were exposed to higher stress conditions. Moreover, no significant sex-specific differences were observed in peak AT strain and hysteresis, indicating that the AT of males did not store and return elastic energy more efficiently than that of females. Thus, the mechanical properties of the AT should be maintained and/or improved through physical exercise.

## 1. Introduction

As the thickest and strongest tendon in the human body, the Achilles tendon (AT) is the key structure that connects the heel and the plantar flexor muscle [1]. The mechanical property of the AT is suitable for storing and releasing elastic energy during walking, running, and jumping [2]. Previous studies have reported a wide range of AT force from 5 kN during single-leg hopping [3] to 9 kN during running [4], which is more than 12 times the body weight (BW). Although the AT can positively adapt to load within a reasonable range during daily activities, excessive and repetitive stress causes tendon degeneration and disease. Therefore, the morphology and mechanical properties of the AT should be investigated for the prevention and treatment of Achilles tendinosis.

Of all the tendons in the foot and ankle joint, Achilles tendinosis is the only disorder that occurs mostly in men. According to previous studies, the men–women ratio of AT rupture ranges from 2:1 to 19:1 [5,6,7]. One possible reason why men are more likely to be exposed to AT tendinosis or ruptures is the physiological differences between sex. The secretion of estrogen in women can promote the synthesis of collagen to maintain the strain of the AT, thereby reducing the incidence of AT injury [8]. Knobloch et al. [9] investigated sex-specific response to eccentric training, and they found that the pain reduction and the improvement in the Foot Ankle Outcome Score and VISA-A score were significantly lower among symptomatic women in contrast to men. This was mainly because the relationship between sex and morphological/mechanical properties of AT remains unclear. Similarly, the previous results of repetitive loading exercises indicated that, compared with the response and recovery to exercise, baseline differences in tendon mechanical properties, which showed less in women, may help to explain the disparity in injury risk [10]. Thus, the sex-specific morphological and mechanical properties of the AT should be explored, which can help to establish sex-specific AT exercise and prevention programs [11,12].

The mechanical properties (e.g., AT force, stress, stiffness) of the AT are affected by muscle activity, joint activity, and characteristics of the tendon and ligament, which are important parameters for evaluating AT maturation, aging, training, and fatigue. However, viscoelasticity, an evident property, was often neglected in previous evaluations of the mechanical properties of the AT [13]. At present, only a few studies have investigated the viscoelasticity of the AT in vivo with ultrasound [13,14,15,16], indicating that the potential mechanism for the reduced risk of AT injury is viscoelastic changes [17]. Compared with the viscous properties, AT with superior elastic properties can store and release elastic energy more efficiently during walking, running, and jumping [13]. Sex, however, tends to be obscured in research. By disaggregating the data of sex in the study of Peltonen et al. [13], who recruited 10 men and 4 women participants, it could be observed that AT hysteresis was 54% greater in men than women. Therefore, it is important to explore the sex differences in the mechanical properties of the AT, otherwise, it may weaken the significance of the results.

The AT can be easily accessible with ultrasound due to its superficiality. In the current study, the AT was loaded on an isokinetic dynamometer synchronized with ultrasound to accurately quantify its mechanical properties. Therefore, this study aimed to investigate the effects of sex on the morphological and mechanical properties (e.g., AT force, stiffness, viscoelasticity) of the AT in inactive individuals. It was hypothesized that sex differences in the morphological and viscoelastic properties of the AT were present; that is, compared with female participants, male participants had (1) longer AT length, larger AT cross-sectional area (CSA), and thicker AT, as well as (2) greater peak plantarflexion torque (PT), peak AT force, peak stress, peak strain, stiffness, and lower hysteresis.

## 2. Materials and Methods

This study was a randomized controlled trial. In total, 30 inactive healthy participants (15 male participants and 15 female participants) were recruited (Table 1). Based on previous data (peak AT strain change between different foot strike runners, d = 0.88) published by Willy et al. [18], a priori power analysis (G*Power 3.0.1, Univ. Kiel, Kiel, Schleswig-Holstein, Germany) determined that 25 participants would power the independent t-test design (α = 0.05 and β = 0.2). All participants were physically inactive and used to wearing flat shoes. They did not meet the scoring criteria of being minimally active in the International Physical Activity Questionnaire Short Form: (i) three or more days of vigorous activity of at least 20 min per day, or (ii) five or more days of moderate-intensity activity or walking of at least 30 min per day, or (iii) five or more days of any combination of walking, moderate-intensity or vigorous-intensity activities achieving a minimum of at least 600 MET-minutes per week [19]. The dominant leg of all participants was the right leg, and there was no lower limb injury in the past year. The female participants recruited were not menstruating and had not taken any form of hormonal contraceptive when participated in the study. No caffeine or alcohol was consumed during the first 2 h of the test, and no strenuous or exhaustive exercise was performed during the first 24 h of the test. Each participant signed an informed consent form approved by the Institutional Review Board of Shanghai University of Sports (No. 102772021RT085) before the experiments.

**Figure 1:**
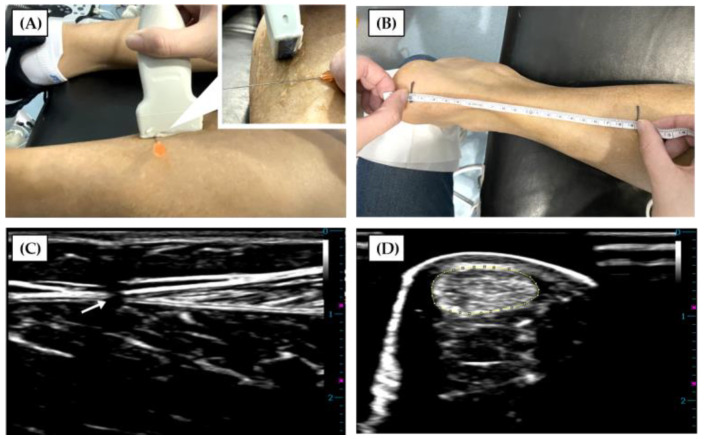
(**A**) The position (myotendinous junction (MTJ) of the medial gastrocnemius (MG) head and Achilles tendon (AT)) was marked on the skin where the ultrasound probe and needle crossed. (**B**) The distance between landmarks, defined as the length of the AT, was measured with tape. (**C**) The black shadow (indicated by a white arrow) in the ultrasound image was where the needle was placed, defined as the MTJ of the MG head and AT. (**D**) The cross-sectional area at the level of the medial malleolus was determined by manually tracing the border of the AT.

Tendon morphological properties were assessed with an ultrasound device (M7 Super, Mindray, Shenzhen, Guangdong, China). The participants were asked to lie prone on a treatment bed with the dominant ankle (defined as the preferred kicking leg [20]) placed in the neutral position (90°). An ultrasound gel was applied at the head of an ML6-15-D probe (10 MHz maximum frequency) at the myotendinous junction (MTJ) of the medial gastrocnemius head and AT. A 25-gauge needle was then inserted between the probe and the skin surface, which was projected in the ultrasound image as a landmark (Figure 1A). Then, the landmark representing the MTJ, where the needle and the ultrasound probe crossed, was marked on the skin (Figure 1C) [21]. The same method was used to determine the insertion point of the AT at the calcaneus. The length of the AT defined as the distance between the MTJ and the insertion point was measured with a tape (Figure 1B). The CSA of the AT was determined by a transverse scan at the level of the medial malleolus with the ultrasound probe placed perpendicular to the AT (Figure 1D) [22].

**Figure 2:**
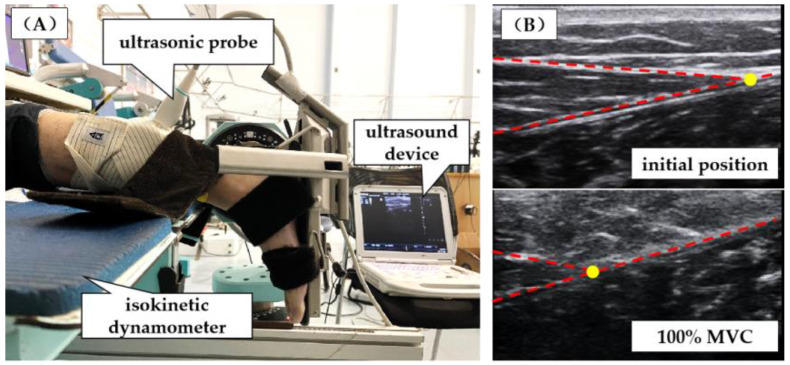
(**A**) Ultrasound probe attached to the dominant leg during the maximum voluntary contraction on the isokinetic dynamometer. (**B**) Ultrasound images of the myotendinous junction of the medial gastrocnemius (MG) head and Achilles tendon (AT). The yellow dot represents the point that is tracked in the image for each frame during the maximum voluntary contraction. To the left of the yellow dot is the MG muscle, and to the right is the AT.

The mechanical properties of the AT were assessed during isometric plantarflexion using an isokinetic dynamometer (CON-TREX MJ, Physiomed, Schnaittach, Freistaat Bayern, Germany). After a 5 min warm-up, the participants were asked to lie prone on a test bench, the waist and thighs were secured by adjustable lap belts and held in position, and the arms fell naturally to the sides. The dominant ankle joint was set at 90° with the knee joint at full extension, and the foot was securely strapped to a footplate connected to the dynamometer. Before measurements, the best maximum isometric voluntary contraction (MVC) force was determined through three trials. For the MVCs, participants were instructed to produce as much force through the ankle plantarflexion as possible. Then, each participant was instructed to develop a gradually increasing force from relaxation to 100% MVC within 5 s, followed by a gradual relaxation within 5 s [15]. During the test, the ultrasonic probe was attached in the sagittal plane at the MTJ with elastic bandages, and the elongation of the AT was obtained (Figure 2). The ultrasonic videos were recorded at 35 Hz, synchronized with an isokinetic dynamometer via an external synchronization box (Biopac Systems Inc., Goleta, CA, USA). Before the test, the participants were given sufficient time to become familiarised with the target tasks.

The thickness and CSA of the AT were analyzed at the level of the medial malleolus using ImageJ software (NIH, Bethesda, MD, USA). The thickness of the AT was measured by the maximum anteroposterior diameter of the tendon, and the CSA of the AT was measured by tracing the surrounding echogenic boundary of the AT. The length of the AT was calculated as the distance between the MTJ and the insertion point. MTJ displacement was tracked from the video with semi-automatic software Kinovea (version 0.8.15). The software requires the user to place one tracking point over the MTJ. The software tracked automatically initially, and then the experimenters confirmed whether the tracking point of each frame was in the correct position—i.e., at the MTJ—and manually adjusted it frame by frame. After tracking, MTJ displacement was output. Tendon elongation was calculated by subtracting the initial tendon length at rest, and the strain was calculated by dividing the elongation by the initial length.

The AT force was calculated by deriving the PT on the dynamometer and moment arm of the AT (MA_AT_):

$$\mathrm{AT}\;\mathrm{force}=\mathrm{PT}\times {\mathrm{MA}}_{\mathrm{AT}}^{-1}$$

where the default value of MAAT is 0.05 m, which is the average vertical distance from the ankle to the anterior border of the AT [23], and the AT force is normalized by the BW.

**Figure 3:**
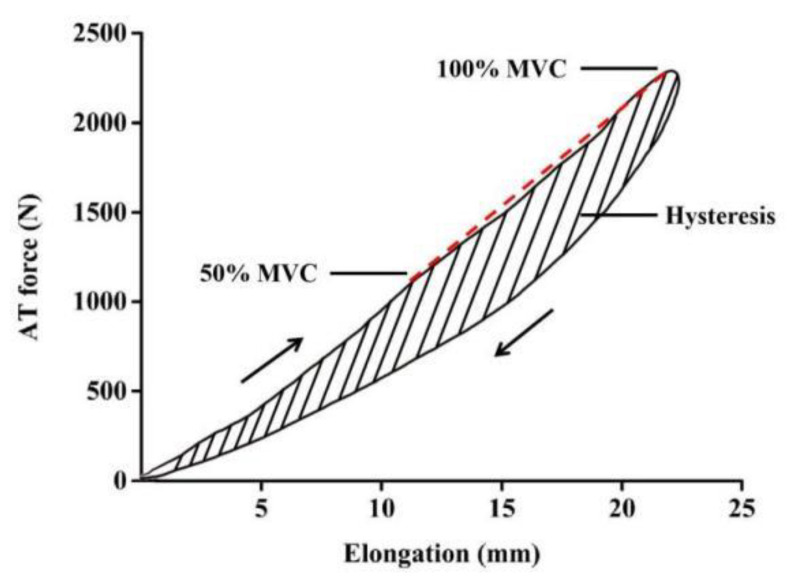
Schematic of tendon stiffness and hysteresis deduction from the Achilles tendon (AT) force–elongation curve. Stiffness (red dashed line) was calculated as the slope of the ascending limb of the AT force–elongation curve at 50–100% maximum voluntary contraction (MVC). Hysteresis was calculated as the area between the ascending and descending limbs.

AT stress was calculated from the AT force divided by the CSA. AT stiffness was calculated as the slope of the least-squares line of the ascending limb of the force–elongation curve between 50% and 100% of MVC force [2]. Hysteresis was calculated by subtracting the area under the descending limb of the force–elongation curve from the area under the ascending limb and dividing the difference by the area under the ascending limb (Figure 3).

All dependent variables were distributed normally as indicated by Shapiro–Wilk tests. Independent t-tests were used to determine the differences in all variables between males and females (25.0, SPSS Inc., Chicago, IL, USA). The 95% confidence intervals and effect size (Cohen’s d) of each parameter in this study was calculated. The significance level was set at 0.05.

## 3. Results

Significant sex-specific differences in the morphological properties of AT were observed. Male participants had 15% longer AT length, 31% larger AT CSA, and 21% thicker AT than female participants (*p* < 0.05, Table 2).

**Figure 4:**
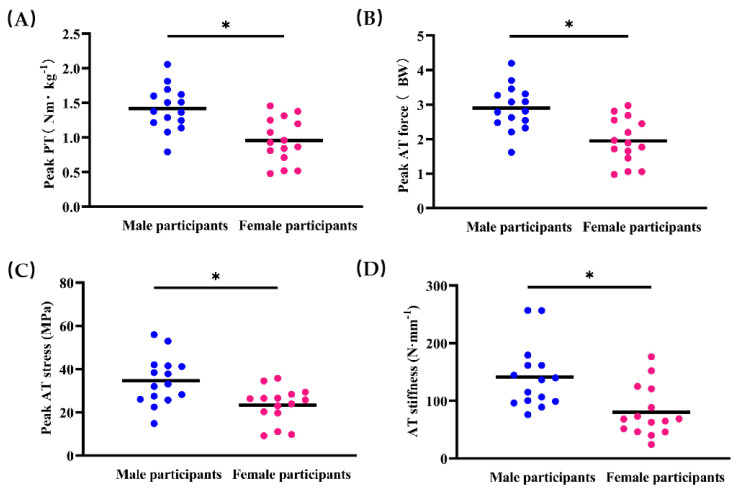
Differences in peak plantarflexion torque (PT, **A**), Achilles tendon (AT) force (**B**), stress (**C**), and stiffness (**D**) between male and female participants. * Indicates *p* < 0.05.

For the mechanical properties of AT, the PT during MVC on the dynamometer was significantly greater in male participants than in female participants (*p* < 0.05, Figure 4A). Compared with female participants, male participants also showed greater peak AT force (Figure 4B), peak AT stress (Figure 4C), and AT stiffness (*p* < 0.05, Figure 4D). However, no significant sex-specific differences were observed in peak AT strain and hysteresis (*p* > 0.05, Table 2).

## 4. Discussion

This study aimed to investigate the effects of sex on the morphological and viscoelastic properties of AT in inactive healthy people. The results showed that compared with female participants, male participants had (1) significantly longer AT length, larger AT CSA, and thicker AT and (2) significantly greater peak PT, peak AT force, peak AT stress, and stiffness, which all supported the hypothesis. However, contrary to the hypothesis, no significant sex-specific differences were observed in peak AT strain and hysteresis. Tendon tissue may be responsive to sex hormones, including progesterone and estrogens, as they possess estrogen receptors [24]. However, the female participants recruited were not menstruating and had not taken any form of hormonal contraceptive when participated in the study. Thus, the sex-specific AT differences observed in this study were largely unaffected by the female sex hormones.

The results of the present study showed that male participants had a longer AT length, greater AT thickness, and larger AT CSA than females. Moreover, the mean AT thickness and mean AT CSA in healthy young male and female participants were 5.1 and 4.2 mm and 60.2 and 45.9 mm^2^, respectively. This finding was similar to the findings of Ying et al. [25], who measured the morphological properties of the AT in 40 healthy Chinese participants (32 men and 8 women) at the medial malleolus level. Using a measurement position consistent with the present study, they observed that the thickness and CSA of the AT of the dominant leg in the inactive group were 5.08 mm and 56.91 mm^2^, respectively. However, the study did not distinguish differences between sex. The present study further found that the thickness and CSA of the AT in male participants were significantly greater than those in female participants. Similarly, Schweitzer [26] found that men had thicker AT via MRI, which also supported our study. The tendons adapted to mechanical loads in daily activities by changing mechanical properties (i.e., stress, strain) and/or morphological characteristics (i.e., CSA), which depend on the intensity of the activity being exposed [27,28]. The AT of men may be subjected to a higher load in daily life.

The peak AT force in male participants was significantly greater than that in female participants. The main reason for this difference was the change of PT during MVC on the dynamometer. This is because the main function of the AT was to transfer triceps surae muscle strength rather than produce force. The results suggested that men had an advantage in triceps surae muscle strength even though inactive people were recruited in this study. However, compared with the average peak AT force of male runners in our previous study (3.4 BW) [29], the inactive group had a significantly lower AT force (2.9 BW in men and 2.0 BW in women). This is mainly because a sedentary lifestyle can lead to poor tendon circulation [30]. Therefore, proper physical exercise is necessary to improve AT properties. In addition, the peak AT stress was significantly higher in male participants than in female participants, which indicated that men may experience higher AT force per unit area. If an increase of stress was more than the range that people could load, the risk of AT injury may increase [31]. This may be one reason why men are more prone to Achilles tendinosis.

This study found that amongst inactive people, male participants of the same age had greater AT stiffness, with a mean value of 141.3 N·mm^−1^, compared to female participants. The results were similar to previous in vivo measurements on the AT (188 N·mm^−1^ during one-legged hopping [14]; 193 N·mm^−1^ at the fast loading rate [13]). According to previous studies, a stiffer tendon is beneficial to sport performances [32]. However, increased stiffness of the tissue with aging also has been proved the decreased ability to withstand repetitive stress, which in turn permitted forceful and sudden contractions to tear the tendon [33]. Up until the present moment, there has been no report of an optimal range of AT stiffness. No consequences may come of naturally stiffer tendon in men for now, yet the potential risk of tendonitis should not be dismissed. As a viscoelastic material, when the stress applied to the AT was removed, the stored mechanical energy did not fully recover. Therefore, the energy recovered is not equal to the energy stored, and the phenomenon of lost energy is known as hysteresis [15]. No sex differences were observed in AT hysteresis, suggesting that both men and women have similar elasticity and viscosity of the AT. This result supported the findings of Sprague et al. [34], wherein the viscoelasticity of the AT is consistent in different sex. The study by Kubo et al. [15] showed that the average hysteresis of the AT in healthy men during MVC was 21%, while people who regularly exercise had lower hysteresis (about 4%) reported by Peltonrn et al. [13]. Low hysteresis is advantageous for the AT because the tendon could store more elastic energy and minimize heat damage. The current consensus view was that lack of exercise may lead to the degeneration of the mechanical properties of the AT [28,35]. Therefore, the mechanical properties of the AT should be maintained and even further improved through physical exercise to reduce the risk of AT injury.

The current study has some limitations. Firstly, how the activity was recorded could only count for some of the likely population variances within a group who self-categorize as inactive. Although the categorical classification of participants based on self-reporting is valid, the nuanced breakdown of sedentary and non-sedentary time in the day may contribute to the variability in your data such that “inactive” participants may vary in terms of daily steps, sedentary time, and work-based physical activity. Secondly, the method of measuring the moment arm of the ankle joint in vivo was a fixed value. The difference between men and women would not be exaggerated by using a fixed value, but the overall in vivo data may be less accurate by a few percent; however, the value obtained in this study was the same as that of Rice et al. [36]. whose participants were similar in terms of age, height, weight to those of this study. Thirdly, the location tested in this study was the junction of the GM and AT. Although this location is consistent with most studies and has good comparability, future studies should consider simultaneously measuring the mechanical properties of the AT at three junctions to comprehensively explore the biomechanical characteristics of the AT. Moreover, future research could consider the role of the menstrual status or hormonal contraceptive in the sex differences on AT.

## 5. Conclusions

The results of this study showed that amongst inactive people, compared with female participants, male participants had significantly longer AT length, larger AT CSA, and thicker AT and greater peak PT, peak AT force, peak AT stress, and stiffness. The findings suggested that in physically inactive adults, the morphological properties of the AT were superior in men, but were exposed to higher stress conditions. Moreover, no significant sex-specific differences were observed in peak AT strain and hysteresis, indicating that the AT of men did not store and return elastic energy more efficiently than that of women. Results of this study indicated the sex differences in the morphological and mechanical properties of AT. A clear understanding of sex differences in the tendon mechanical characteristics would enable further generating sex-specific treatment strategies of Achilles tendinopathy in the clinic.

## Figures and Tables

**Table 1:** Participant characteristics (mean ± SD).

Group	Age (Years)	Height (cm)	Body Mass (kg)
Male participants (*n* = 15)	24.7 ± 2.6	173.7 ± 3.6	70.8 ± 7.1
Female participants (*n* = 15)	24.1 ± 2.7	161.5 ± 3.7	54.6 ± 8.3
*p*-value	0.538	<0.001	<0.001

**Table 2:** Morphological and mechanical properties of Achilles tendon (AT) in male and female participants.

Variables	Male Participants (*n* = 15)	Female Participants (*n* = 15)	*p*-Value	95% Confidence Interval	Cohen’s d
AT length (cm)	20.4 ± 2.5	17.7 ± 2.2	0.005	[0.84628; 4.38038]	1.15
AT CSA (mm^2^)	60.2 ± 10.5	45.9 ± 8.6	<0.001	[7.24997; 21.57403]	1.49
AT thickness (mm)	5.1 ± 0.6	4.2 ± 0.4	<0.001	[0.56271; 1.35196]	1.77
Peak plantarflexion torque (Nm·kg^−1^)	1.4 ± 0.3	1.0 ± 0.3	<0.001	[0.22808; 0.70413]	1.33
Peak AT force (BW)	2.9 ± 0.7	2.0 ± 0.7	<0.001	[0.46547; 1.43700]	1.29
Peak AT stress (MPa)	34.7 ± 11.2	24.7 ± 8.6	0.004	[3.98736; 18.64666]	1.00
Peak AT strain (%)	9.7 ± 2.2	10.5 ± 2.5	0.349	[−2.54486; 0.93004]	0.34
AT stiffness (N·mm^−1^)	141.3 ± 55.6	80.7 ± 43.8	0.003	[23.18262; 98.10117]	1.21
Hysteresis (%)	33.1 ± 13.4	27.6 ± 12.5	0.252	[−4.17099; 15.25145]	0.42

## Data Availability

The raw data supporting the conclusions of this article will be made available by the authors upon reasonable request.
